# Editor's Message

**DOI:** 10.29173/jchla29648

**Published:** 2022-12-01

**Authors:** Colleen Pawliuk

Welcome to the December issue of JCHLA/JABSC! As incoming Editor-in-Chief I want to first thank Alanna Campbell, former Editor-in-Chief, who led the successful effort to have JCHLA/JABSC indexed in PubMed Central. I also want to welcome the new members to the Editorial Board: Jessica McEwan, Junior Editor, and Meredith Fischer, Copyeditor.

In our continued work to educate ourselves on topics related to Equity, Diversity, and Inclusion (EDI), the Editorial Board met in November to discuss the webinar “Driving diversity, equity, and inclusion to shape the future of publication ethics” presented by the Committee on Publication Ethics (COPE). The focus of our meeting was on how to bring what we learned from the speakers into our practice in each of our roles and to JCHLA/JABSC as a whole. The discussion sparked several ideas for how we can take action to make JCHLA/JABSC more inclusive for our authors, readers, reviewers and Editorial Board members. I hope to share more detail of this work in the coming months. I also want to continue the call to encourage our authors to consider submitting their work related to EDI to JCHLA/JABSC.

This issue we have the JCHLA/JABSC Student Paper Prize winning article by Vinson Li, which uses bibliometric methods to map the field of health science librarianship research. Also, in this issue are two books reviews, *Academic libraries and collaborative research services* and *Communities of practice in the academic library: strategies for implementation*.

We hope you enjoy the December issue!

**Colleen Pawliuk**

*JCHLA/JABSC Editor-in-Chief*

*Email: editor@chla-absc.ca*

Bienvenue au numéro de décembre de JABSC / JCHLA ! En tant que nouvelle rédactrice en chef, je tiens tout d’abord à remercier Alanna Campbell, l’ancienne rédactrice en chef, qui a mené à bien les efforts visant à indexer JABSC / JCHLA dans PubMed Central. Je tiens également à souhaiter la bienvenue aux nouvelles membres du comité de rédaction : Jessica McEwan, rédactrice en chef adjointe, et Meredith Fischer, correctrice.

Dans le cadre de notre travail continu d’éducation sur les sujets liés à l’équité, la diversité et l’inclusion (EDI), le comité de rédaction s’est réuni en novembre pour discuter du webinaire «Driving diversity, equity, and inclusion to shape the future of publication ethics» présenté par le Committee on Publication Ethics (COPE). Notre réunion s’est concentrée sur la façon de mettre en pratique ce que nous avons appris des conférenciers et conférencières dans chacun de nos rôles et au sein du JABSC / JCHLA dans son ensemble. La discussion a suscité plusieurs idées sur la façon dont nous pouvons agir pour rendre JABSC/JCHLA plus inclusif pour nos auteurs et autrices, notre lectorat, nos réviseurs et réviseures et les membres du comité de rédaction. J’espère partager plus de détails sur ce travail dans les mois à venir. Je souhaite également continuer à encourager nos auteurs et autrices à envisager de soumettre leurs travaux relatifs à l’EDI au JABSC / JCHLA.

Dans ce numéro, nous présentons l’article de Vinson Li, qui a remporté le prix de l’article étudiant du JABSC / JCHLA, et qui utilise des méthodes bibliométriques pour cartographier le domaine de la recherche en bibliothéconomie des sciences de la santé. Vous trouverez également dans ce numéro deux comptes rendus de livres, *Academic libraries and collaborative research services* et *Communities of practice in the academic library : strategies for implementation*.

Nous espérons que vous apprécierez ce numéro de décembre !

**Colleen Pawliuk**

*Rédactrice en chef, JABSC / JCHLA*

*Courriel: editor@chla-absc.ca*

